# Preparation Method and Performance Evaluation of a Gel Based on AM/AMPS Copolymer

**DOI:** 10.3390/gels8120802

**Published:** 2022-12-07

**Authors:** Yunling Ran, Guicai Zhang, Ping Jiang, Haihua Pei

**Affiliations:** School of Petroleum Engineering, China University of Petroleum (East China), Qingdao 266580, China

**Keywords:** AM/AMPS copolymer, high temperature and high salinity resistant gel, phenolic gel, plate model experiment

## Abstract

Polymer gels have been widely used in high water cut oilfields for profile control and water plugging. It is urgent to develop a gel suitable for the Tahe Oilfield (Temperature: 130 °C, salinity: 2.2 × 10^5^ mg/L) in China. A stable gel was prepared by using an acrylamide (AM)/2-acrylamide-2-methyl propanesulfonic acid (AMPS) copolymer crosslinked with urotropin (HMTA), hydroquinone (HQ), thiourea and Nano-SiO_2_. This paper covers a step-by-step process for designing gels based on experience with preparing gels. A wide range of combinations between polymers and crosslinking agents with and without stabilizers were investigated, and the results indicated that there is an optimal value of AMPS content of AM/AMPS copolymers in the preparation of gels. Increasing the mass fraction of copolymer and using stabilizer enhanced the performance of gel, but an excessive amount of crosslinking agent was not conducive to the stability of gel. The work optimized the formula of plugging agent suitable for the high temperature and high salt (HTHS) condition in the Tahe Oilfield. The gelling solution had a long gelation time of 20 h. The gel had high strength (Sydansk’s gel-strength code of “G”) with storage modulus of 12.9 Pa and could be stable for half a year at 130 °C and 2.2 × 10^5^ mg/L of salinity. The plate model that could be heated and pressurized was used to simulate the oil flooding and profile modification under the condition of the Tahe Oilfield for the first time. The experiment results showed that the oil recovery could be increased by 13.22% by subsequent water flooding under heterogeneous formation condition. Therefore, it was fully confirmed that the plugging performance of AM/AMPS phenolic gel prepared in the work was excellent. The information provided in the study could be used as a reference for the design and evaluation of polymer gels in other HTHS reservoirs.

## 1. Introduction

Water production from oil wells is a common problem in oilfield development [1,2]. Due to the heterogeneity of the formation, the displacement medium (water, gas, steam, etc.) often preferentially forms a dominant channel in the high permeability formation. With the development of the oilfield, the low permeability reservoir is rarely developed [3]. To solve this problem, it is necessary to effectively block the high flow passage and force the displacement medium to turn to the reservoir with low permeability to increase the sweep efficiency of the displacement medium. At present, the methods used mainly include polymer flooding, foam flooding, gel, etc. [4,5,6]. Polymer gels have been successfully applied in high water cut oilfields as a profile control and water shutoff agent [7,8,9]. However, there are still many technical problems when polymer gels are applied to the HTHS reservoirs such as the Tahe Oilfield [8,10]. The gels prepared from partially hydrolyzed polyacrylamide (HPAM) are often weak in gel strength and short in effective plugging period in the HTHS reservoirs. Therefore, the preparation of temperature and salt resistant gels has become a research hotspot.

Moradi et al. [7] prepared a gel which could be stable for 2.5 years at 113 °C in seawater. The gel was based on HPAM solution and added phenol and formaldehyde as crosslinking agents. Eriksen et al. [9] added phenol and HMTA to the AM/AMPS copolymer with 40% AMPS content in the brine of the North Sea, and the prepared gel could be aged at 120 °C for 30 days without dehydration. Juarez et al. [11] compared the stability of chromium gel, polyethyleneimine gel, and phenolic gel formed by copolymers with different AMPS contents in salt water with a temperature of 100 °C and salinity of 5951 mg/L. The experiment showed that copolymers with appropriate AMPS content usually formed more stable gels. Liu et al. [10] used AM/AMPS/NVP polymer, bisphenol A and formaldehyde to prepare phenolic gel in distilled water, which was injected into saline water with a salinity of 200,000 mg/L, the properties of the gel remained stable after 30 days at 150 °C.

Zhu et al. [12] prepared a gel with 0.6 wt% AM/AMPS/NVP, 0.6 wt% resorcinol, 0.6 wt% HMTA, 0.05 wt% thiourea, and 0.05 wt% cobalt salts, respectively. At 150 °C, the gel was dehydrated 20% after aging 23 days in salt water with a salinity of 116,880 mg/L, while 60% of the water was dehydrated after aging 30 days in salt water with a salinity of 233,760 mg/L. Unomah et al. [13] prepared gels with HPAM and five other copolymers with different sulfonate contents in salt water at 110 °C. It was found that AM/AMPS gels were significantly better than HPAM gel under HTHS conditions.

Dai et al. [14] reported that a gel reinforced by nano-SiO_2_ could withstand a temperature of 110 °C and a salinity of 212.6 g/L. However, if the molecular weight of the copolymer is too large, its dissolution in water would lead to entanglement and aggregation, which would often affect the gel structure and lead to a nonuniform gel network [15]. Some studies also showed that the entanglement state of polymer in water could be changed by adding appropriate nanoparticles in polymer aqueous solution [15,16,17,18]. Ginzburg et al. [17] found that, when the particle size of the nanoparticles was smaller than the rotation radius of the polymer chain, the interaction between the polymer chains could be inhibited.

Physical simulation technology is one of the key technologies in indoor research. In the process of EOR research, physical simulation experiments are required to complete the oil displacement mechanism, formulation, and injection mode optimization performance evaluation of oil displacement agent and its effect verification. At present, the plugging performance evaluation of gels is mostly carried out in the sand pack [19]. In this paper, the plate model is used for reservoir simulation for the first time. Compared with the sand pack, the plate model could be pressurized and heated, which means that it could simulate the real reservoir conditions more effectively.

In a word, although many scholars have synthesized polymer gels including the AM/AMPS copolymer by adding various cross-linking agents, the experimental temperature and formation water salinity are quite different from the conditions of the Tahe Oilfield. At present, there is not sufficient analysis and evaluation of temperature and salt resistant gels. In this paper, by taking gelation time, strength, and thermal stability of gels as evaluation criteria, a gel suitable for the HTHS conditions in the Tahe Oilfield was prepared. In addition, the temperature resistant and pressure resistant plate model used for the first time can more truly simulate the reservoir conditions. The gel plugging test shows that the prepared gelling fluid can form a gel with high strength and good stability under the conditions of the Tahe reservoir. The secondary water drive can improve the recovery by more than 13%.

## 2. Results and Discussions

### 2.1. Formula Optimization of Temperature and Salt Resistant Phenolic Gel

#### 2.1.1. Effect of the AM/AMPS Copolymer Mass Fraction on the Properties of Gel

**Figure 1:**
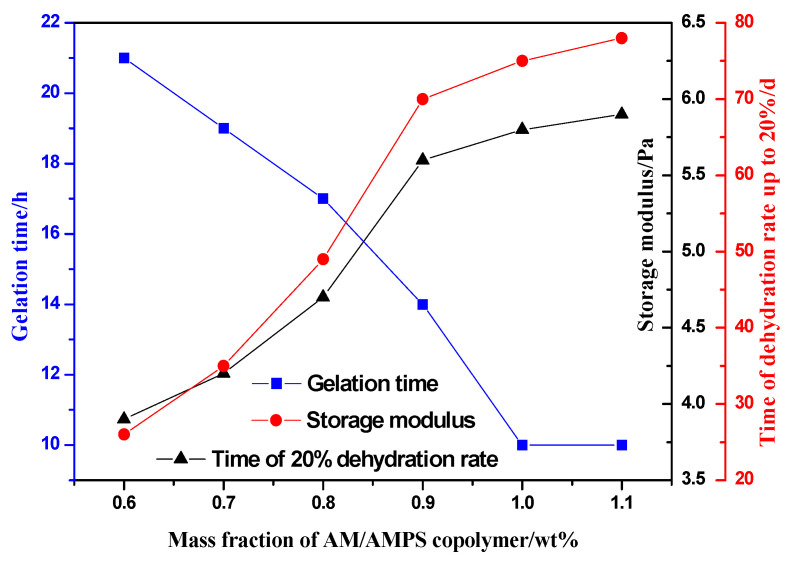
Effect of AM/AMPS copolymer mass fraction on the properties of gel.

The effects of the AM/AMPS copolymer mass fraction on gelation time, gel strength, and long-term stability of gel samples were studied at 130 °C. The AMPS content was fixed at 50 mol%, and 0.1 wt%HQ and 0.2 wt% HMTA were used as crosslinking agents. The mass fraction of the AM/AMPS copolymer used to prepare the gel ranged from 0.6 wt% to 1.1 wt%. The results are shown in Figure 1.

It can be seen from Figure 1 that, as the mass fraction of the copolymer increases from 0.6 wt% to 1.1 wt%, the gelling time gradually shortens from 21 h to 10 h, the gel strength increases, and time of dehydration rate up to 20% is extended from 26 d to 84 d, which means the gel stability is significantly enhanced. Due to the fact that the largest composition of the gelling solution is solvent (usually more than 98 wt%), the gels tend to dehydrate. When the elastic potential of the gel is greater than the mixing potential, the gel will contract to balance the two potentials, resulting in the expulsion of the solvent from the gel network. Dehydration usually occurs at relatively low polymer concentrations because the gel has a lower mixing potential than at higher polymer concentrations [20,21]. Gales et al. [20] have found that a high polymer mass fraction in a gel formulation may result in a high free energy of mixing, which reduces the difference between the free energy of mixing and the free energy of elasticity and reduces the rate of dehydration. In addition, many studies have demonstrated that increasing polymer mass fraction can effectively improve gel properties [22,23].

It can be explained that the increase of the mass fraction of the copolymer increases the number of amide groups, increasing the cross-linking points. The cross-linking reaction between the copolymer and the cross-linking agent occurs more easily, which shortens the gelling time; at the same time, the strength and stability of the gel are also improved. When the mass fraction of the copolymer exceeds 0.9 wt%, the continuous increase has little effect on improving the stability of the gel. The reason may be that the 0.9 wt% copolymer basically reacts completely with the quantitative crosslinking agent. If the mass fraction of the polymer is increased, the “excess” copolymer can’t be effectively crosslinked. At the same time, considering that the increase of the polymer will increase the cost of the gel, the preferable AM/AMPS copolymer mass fraction is 0.9 wt%.

#### 2.1.2. Effect of AMPS Content on the Properties of Gel

**Figure 2:**
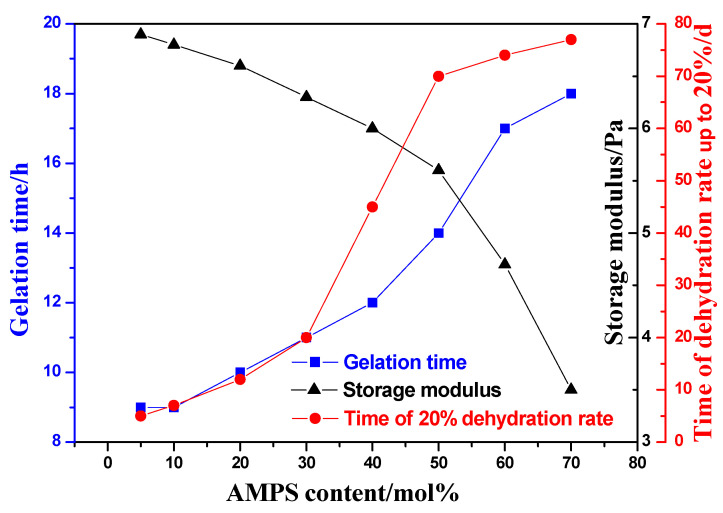
Effects of AMPS content on properties of gels.

With the mass fraction of copolymer set at 0.9 wt%, the HQ at 0.1 wt%, and the HMTA at 0.2 wt%, the influence of AMPS content in the copolymer on the gel properties was investigated at 130 °C. The experimental results are shown in Figure 2.

It can be seen that, with the increase of AMPS content in the copolymer, the time of dehydration rate up to 20% gradually increases. On one hand, the sulfonic acid group on the AMPS monomer is not sensitive to salt, which weakens the dehydration effect of inorganic salt, and the polymer chain can be relatively stretched in the Tahe simulated water; On the other hand, AMPS monomers contain large side groups, which increase the rigidity of polymer molecules and inhibit the hydrolysis of amide groups to a certain extent. Therefore, gels with high AMPS content have better stability. With the increase of AMPS content in the copolymer, the content of AM decreases correspondingly. When the mass fraction of copolymer is fixed, the number of crosslinking points provided by the copolymer decreases, and the formed crosslinking network is loose. Therefore, the gel strength decreases, and the gelation time increases as AMPS content increases, especially while the AMPS content is greater than 50%. Zhang et al. [24] have also found that there is an optimal value of AMPS content in polymers when gels form. The copolymer of 30% AMPS content was chosen in the follow-up experiments. This is because the experimental condition (Temperature: 130 °C, salinity: 71,695 mg/L) is different from the Tahe Oilfield. The copolymer with 50% AMPS content is more conducive to improving the salt tolerance than that with 30% AMPS content in the Tahe simulated water.

To sum up, although the increase of AMPS content makes the gel more stable, the increase of AMPS content will increase the cost of the gelling solution. In addition, the gel used as a plugging agent must reach a certain strength. Therefore, the AM/AMPS copolymer with 50 mol% AMPS content is selected to prepare temperature and salt resistant gel.

#### 2.1.3. Optimization of Phenolic Cross-Linking Agent

- Optimization of crosslinking agent types

Phenolic gel is the most commonly used temperature and salt resistant gel at present. The commonly used phenols include HQ, resorcinol, phenol, etc. The commonly used aldehydes include formaldehyde, HMTA, etc. The mass fractions of phenol and aldehyde crosslinkers were set to 0.1 wt%. The effects of the types of phenol and aldehyde crosslinkers on gelling time, gelation strength, and long-term stability of the system were investigated at 130 °C. The experimental results are shown in Table 1 and Table 2.

If 0.1 wt% HMTA is used as the fixed aldehyde cross-linking agent and HQ, resorcinol, and phenol are used as the phenol cross-linking agents, respectively, the experiment shows that there is little difference in gel strength. The shortest gelation time of the HQ and HMTA system is 15 h, and the time of dehydration rate up to 20% is after 70 days, while the longest gelation of resorcinol and HMTA system is 360 h, and the time of dehydration rate up to 20% is after 32 days. It is believed that HQ can form an intermolecular hydrogen bond in aqueous solution, and its structure is more stable than that of phenol at high temperature; the steric hindrance effect of resorcinol is far greater than that of HQ and phenol, so the gelling time of the resorcinol system is far greater than that of the phenol and HQ system.

With the total concentration of phenol crosslinking agent set at 0.1 wt% and HQ mixed with phenol and resorcinol, respectively, it is found that, compared with a phenol or resorcinol single system, the gelling time of the mixed system shortens, and the gel stability improves, but the stability of the mixed system is not better than that of the HQ single system.

If the fixed phenol cross-linking agent is 0.1 wt% HQ, the aldehyde cross-linking agent is p-benzaldehyde, m-benzaldehyde, and HMTA, respectively. The experiment shows that the m-benzaldehyde & HQ system and p-benzaldehyde & HQ system does not form gel. This is because HMTA will decompose into small molecules of formaldehyde under high temperature conditions, while p-benzaldehyde and m-benzaldehyde have larger steric hindrance effect compared with formaldehyde, so it is difficult to be cross linked with copolymer. As a result, HQ and HMTA were selected as the crosslinking agents for experimental research.

- Optimization of crosslinking agent concentration

**Figure 3:**
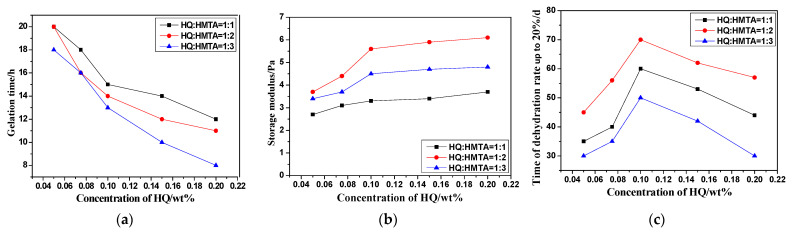
Effect of cross-linking agent with different proportions on gel properties. (**a**) Effect of cross-linking agent with different proportions on gelation time; (**b**) Effect of cross-linking agent with different proportions on gel strength; (**c**) Effect of cross-linking agent with different proportions on long-term stability.

After setting the content of AMPS monomer in the AM/AMPS copolymer as 50 mol%, and the mass fraction of the copolymer as 0.9 wt%, the effects of different mass fractions and proportions of HQ and HMTA on the gelling time, gel strength, and long-term stability of the system were investigated at 130 °C with 0.9 wt% copolymer. The experimental results are shown in Figure 3.

It can be seen that for HQ and HMTA with different mass fractions and proportions, the gelling conditions of the gel system are different:

Firstly, with the increase of the crosslinker, the gelling time decreased, but the difference was not significant; the storage modulus of gel increased gradually;

Secondly, with the increase of the cross-linking agent, the stability of the gel will firstly increase and then decrease. The result indicates that, when the concentration of the polymer is constant, the number of its cross-linking sites is limited. Before the cross-linking sites on the polymer molecular chain reach the saturation state, with the increase of the concentration of the cross-linking agent, the polymer molecules are tightly bound to form a gel with high strength. When the mass fraction of HQ exceeds 0.1 wt%, the stability of the system will be significantly reduced due to the over crosslinking phenomenon [25];

Thirdly, among the phenolic cross-linking agent systems, the gel formed by the HQ: HMTA = 1:2 system has the highest strength and the best stability. If the amount of HMTA is insufficient, the sites on the polymer molecular chain are not adequately crosslinked; while the amount of HMTA is too high, it will decompose into formaldehyde in the aqueous solution, which will be detrimental to the gel.

Therefore, HQ: HMTA = 1:2 was selected as the ratio of phenolic cross-linking agent, and the concentration was 0.1 wt% and 0.2 wt% in subsequent experiments, respectively.

#### 2.1.4. Optimization of Stabilizer

- Optimization of type and mass fraction of deoxidizer

Because acrylamide polymers are prone to oxidative degradation at high temperatures (above 90 °C), a certain amount of deoxidizer should be added to the gel system to inhibit it.

At present, the commonly used deoxidizers in high temperature gel are sodium nitrite, sodium thiosulfate, thiourea, etc. The concentration of polymer, HQ and HMTA were identical among different gel samples, which were fixed at 0.9 wt%, 0.1 wt%, and 0.2 wt%, respectively. The effects of the type of deoxidizer with the same mass fraction on the gelling time, gel strength, and long-term stability of the system were investigated at 130 °C. The experimental results are shown in Table 3. It can be seen that, compared with sodium sulfite and sodium nitrite, the gelling system with thiourea has the shortest gelling time, the highest gelling strength, and the best stability, which indicates that thiourea has better deoxygenation effect under HTHS conditions.

The concentration of polymer, HQ and HMTA were identical among different gel samples, which were fixed at 0.9 wt%, 0.1 wt% and 0.2 wt%, respectively. The effects of thiourea with different mass fractions on the gelling time, gel strength, and long-term stability of the system were investigated at 130 °C.

**Figure 4:**
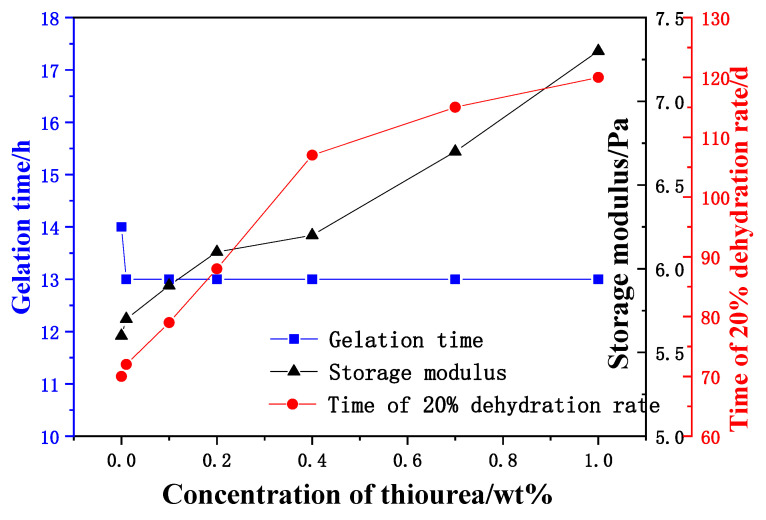
Effect of Thiourea Concentration on Gel Properties.

Thiourea with different mass fractions has little effect on the gelling time and strength, as shown in Figure 4. When there is no thiourea in the gel, the dehydration rate in 70 days is equal to 20%. With the increased concentration of thiourea in the gel, the stability of the gel is enhanced, indicating that thiourea can reduce the gel dehydration rate by inhibiting the oxidative degradation of AM/AMPS copolymer. When the mass fraction of thiourea is more than 0.4 wt%, the increase rate when the gel dehydration rate is equal to 20% slows down. Therefore, the mass fraction of thiourea is optimized to be 0.4 wt%. The research of Chen et al. [26] found similar results, that the effect of thiourea on stabilizing gel would not be enhanced when it reached a certain concentration.

- Effect of nano-SiO_2_ on properties of gel

**Figure 5:**
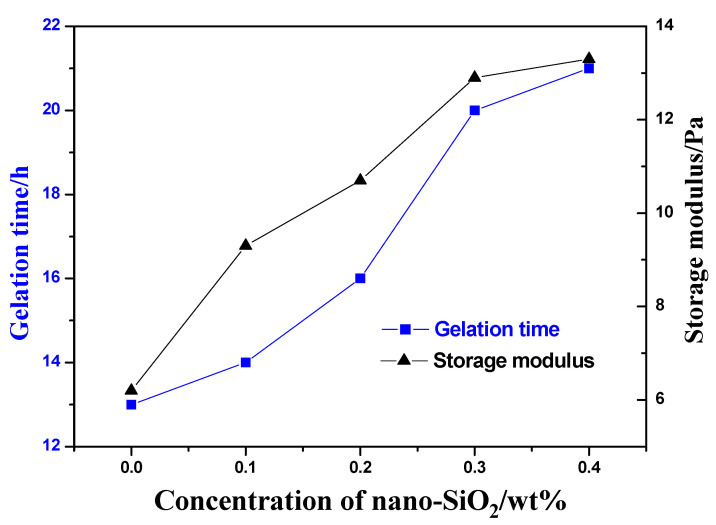
Gel strength and thermal stability of different concentrations of Nano-SiO_2_.

Nano-SiO_2_ particles were added into the gel system to study its influence on gel forming performance under the Tahe reservoir conditions. Gel samples with different concentrations of Nano-SiO_2_ were prepared. The reference gel system comprises 0.9 wt% AM/AMPS copolymer, 0.1 wt% HQ, 0.2 wt% HMTA, and 0.4 wt% thiourea. The experimental results are shown in Figure 5.

It can be found that the storage modulus of the gel without Nano-SiO_2_ is only 6.2 Pa and increases to 9.3 Pa after 0.1 wt% Nano-SiO_2_ are added. When the concentration of Nano-SiO_2_ increases to 0.3 wt%, the storage modulus is about 2 times higher than that of the gel without nanoparticles. Therefore, the strength of the gel can be improved by adding Nano-SiO_2_. In addition, the time of dehydration rate up to 20% of gel without Nano-SiO_2_ is within 107 days at 130 °C, while the gel with 0.3 wt% Nano-SiO_2_ could be stable for half a year. When 0.4 wt% Nano-SiO_2_ was added, the gelation time became longer, but the gel properties were not significantly enhanced.

The addition of Nano-SiO_2_ can effectively inhibit the dehydration of the gel, because the nanoparticles could interact with the dipole by adsorbing water molecules (or hydrated ions) through hydrogen bonds, which could improve the water holding capacity of the gel. Nano particles can also form hydrogen bonds with amide groups [27,28,29,30], significantly delaying the hydrolysis of amide groups and increasing the network density of gel, which is the main reason for nanoparticles to improve the strength and stability of gels.

**Figure 6:**
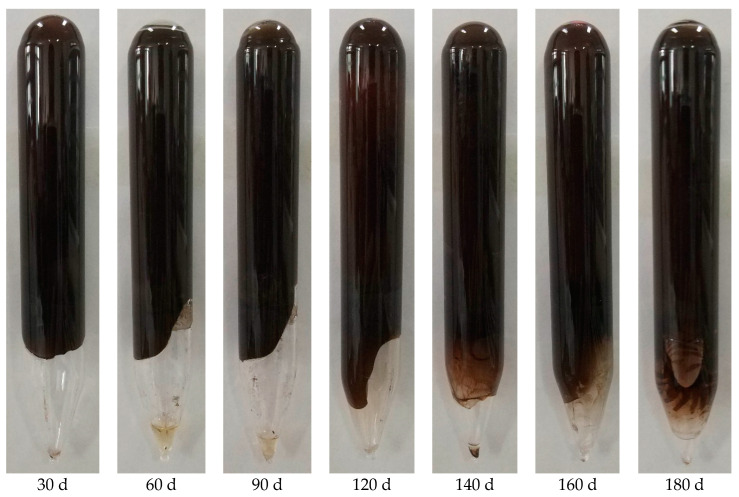
Pictures of gel samples at different heat treatment time.

Based on the above studies, the optimal gel formulation was finally selected: AM/AMPS copolymer with 0.9 wt% mass fraction and 50 mol% AMPS content, 0.1 wt% HQ, 0.2 wt% HMTA, 0.4 wt% thiourea, and 0.3 wt% nano-SiO_2_. The prepared gel is shown in Figure 6. It can be found that the prepared gel has good stability in half a year indicating its great application potential.

### 2.2. Performance Evaluation of the Prepared Gel

#### 2.2.1. Evaluation of Gel in Laboratory

**Figure 7:**
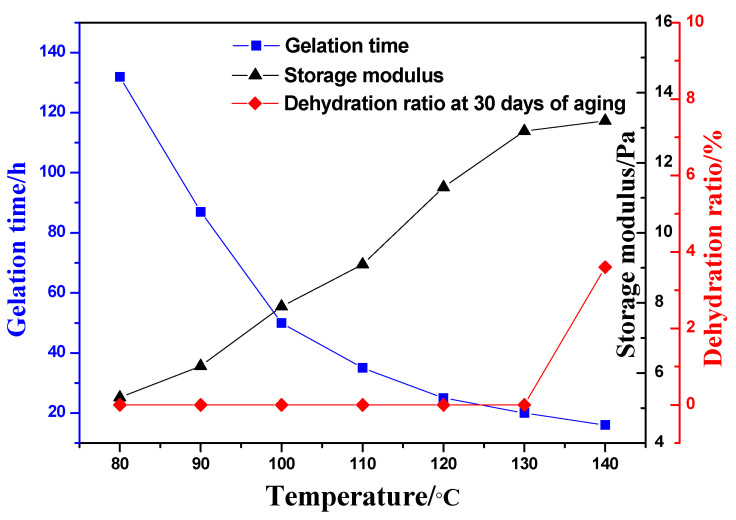
Gelling performance of gelling solution at different temperatures.

The gelling properties of the optimized gel formulation were investigated at different temperatures, ranging from 80 °C to 140 °C. The results are shown in Figure 7. With the increase of temperature, the gelling time of the gel shortened, and the gel strength increased. No dehydration occurs within 30 days when the temperature does not exceed 130 °C, while the dehydration rate within 30 days is only 3.6 wt% at 140 °C. Therefore, the gel is suitable for use in reservoirs of 130 °C.

#### 2.2.2. Blocking Simulation Using Two-Dimensional Plate Model

**Figure 8:**
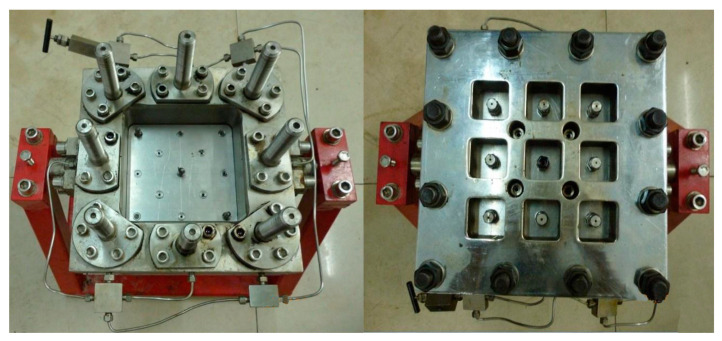
Two-dimensional plate model used in the experiment.

The two-dimensional plate model was designed by authors and made by the petroleum research Co. LTD, Jiangsu, China. The model is shown in Figure 8; the size of the plate model is 15 cm × 15 cm, sand filling thickness is about 3 cm. When filling the model, the upper cover can be opened and tightened with nuts after filling. The confining pressure of the plate model can be adjusted by 8 confining pressure columns.

Under the real geological conditions, the reservoir is basically heterogeneous. The plugging performance of gel under the conditions of the Tahe reservoir was investigated by using the two-dimensional plate model with a 3.0 cm wide high permeability zone along a diagonal direction on the plane, which is filled with 40–50 mesh quartz sand; the rest of the model is the low permeability zone filled with 100–120 mesh quartz sand. A total of 2 MPa confining pressure on the model was added, and the test system was set at a pressure of 0.3 MPa to maintain the water in a liquid state at high temperatures.

**Figure 9:**
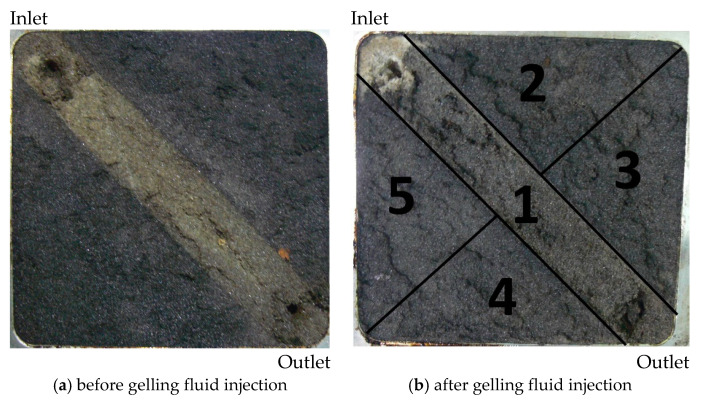
Water drive effect before and after gelling fluid injection (heterogeneous plate model).

After the plate model was saturated with water and oil in order, the oil displacement and water plugging test was carried out. The specific experimental parameters and details are shown in the experimental methods in Section 4.2.3. The comparison of water drive effects before and after the injection of gelling fluid is shown in Figure 9. It can be seen from Figure 9a that, when water is injected into heterogeneous reservoirs, water will preferentially form channeling from the high permeability strip. After water cut reaches 93.74%, the oil content in the oil sand should be low because the color of the high permeability area is obviously lighter. A total of 0.3 PV gelling fluid was displaced to the middle of the model with displacement fluid, gelation was awaited at 130 °C for 24 h, and water was continually injected until the water cut was 98%. Figure 9b shows that the gelling fluid forms a strong gel in the middle of the model, which plays a plugging role. In front of the profile control zone, a large area of oil sand turns white, while a small part of oil sand turns white in the non-high permeability strip after the profile control zone. This is because the formation of gel blocks the “dominant channel” in the high permeability strip, causing water to bypass, flowing to the low permeability part and displacing the oil in the oil sand. The color of oil sand becomes lighter, which indicates that the injection of gelling fluid improves the sweep efficiency during water injection development. In addition, in order to observe and record the appearance of the oil sand in the model, as shown in Figure 9b, the oil sand is divided into five parts and numbered as 1–5.

**Figure 10:**
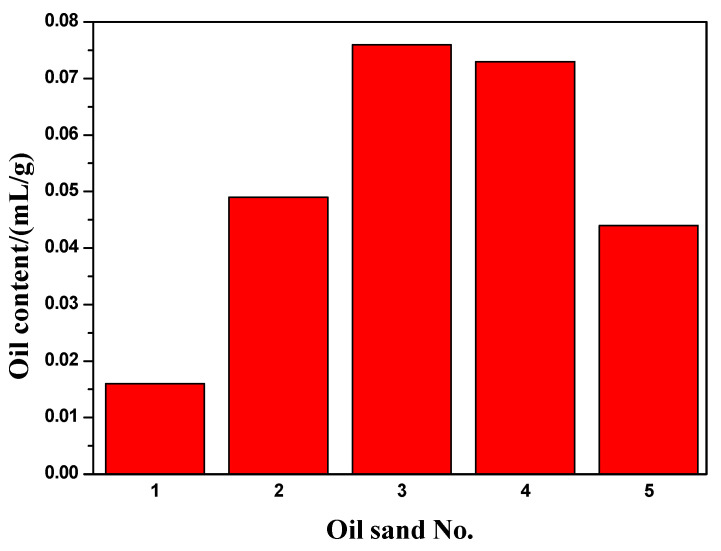
Oil content of oil sand in different parts of heterogeneous plate model.

After the dry distillation test, the oil content per gram of oil sand in different parts is shown in Figure 10. It can be seen from the figure that the oil content in the oil sand decreases. The oil saturation of the high permeability strip is relatively low, which is mainly due to the formation of water channeling in the high permeability strip before the injection of gelling fluid, which results in less residual oil.

**Figure 11:**
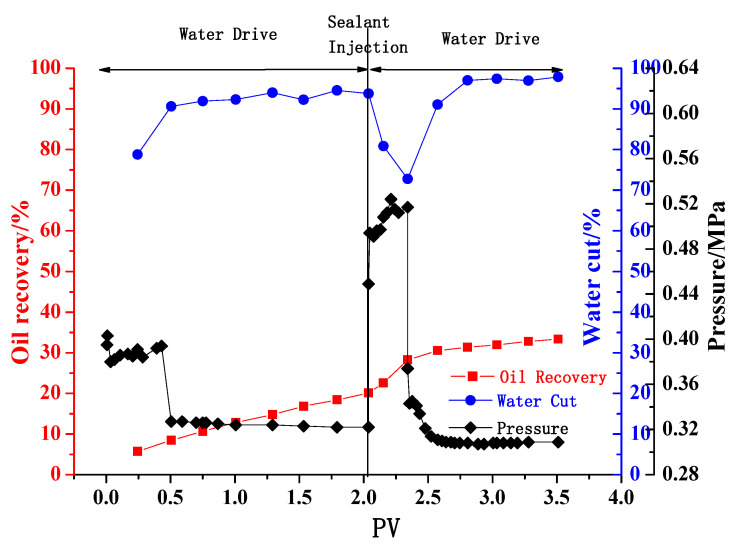
Production curve of heterogeneous model.

Figure 11 shows the changes of oil recovery, water cut and inlet pressure in the experiment. It can be seen that, with the increase of water injection volume, the recovery gradually increases. When the water cut in the produced liquid reaches 93.93%, the cumulative oil production is 36.8 mL, and the recovery is 20.11%. After the gelling fluid is injected, the gel fills part of the pores in the model, and the inlet pressure increases sharply. After a new water drive path is formed, the inlet pressure decreases sharply, the recovery gradually increases, and the water cut decreases. When the water cut in the produced fluid reaches 97.87%, the cumulative oil production is 61.0 mL, and the recovery reaches 33.33%. That is to say, the oil recovery of the heterogeneous model can be increased by 13.22% after the gel is added.

In a word, the gelling fluid has formed a strong gel in the reservoir, which had played a good plugging effect. The secondary water drive could improve the oil recovery by 13.22%, which fully confirms the effectiveness of the AM/AMPS phenolic gel prepared in this paper. The PV number, injection location, and gel injection time will be further studied in the follow-up work.

## 3. Conclusions

1. The compositions of gelling fluid have great influence on the properties of gel. There is an optimal value of AMPS content of AM/AMPS copolymers in the preparation of gels. Increasing the mass fraction and using stabilizer can enhance the performance of gel, but an excessive amount of crosslinking agent is not conducive to the stability of gel.

2. The formula of temperature and salt resistant gel was optimized: 0.9 wt% AM/AMPS copolymer, 0.1 wt% HQ, 0.2 wt% HMTA, 0.4 wt% thiourea, and 0.3 wt% nano-SiO_2_. With this formula, a high strength gel (Sydansk’s gel strength code of “G”) with gelation time of 20 h, gel strength of 12.9 Pa, and long-term thermal stability of up to 6 months was prepared.

3. The gelling performance of the gel formulation system was investigated in the heated and pressurized plate model. The results show that the gelling fluid can form high strength gel under the conditions of simulating the Tahe Oilfield, effectively block the high seepage channel, and improve the recovery by more than 13%.

## 4. Materials and Methods

### 4.1. Materials

The AM/AMPS copolymer (molecular weight of about 6 million g/mol) was provided by Qingdao Mengjia Chemical Co., Ltd.; NaCl, CaCl_2_, MgCl_2_, NaHCO_3_ etc. were purchased from Sinopharm Chemical Reagent Co., Ltd. (Shanghai, China); Thiourea, sodium nitrite, sodium thiosulfate, HQ and HMTA etc. were purchased from Shanghai Aladdin Biological Technology Co., Ltd. (Shanghai, China); Nano-SiO_2_ with 30 wt% solid content was purchased from Sigma Aldrich, and the median particle size is about 10 nm.

The total dissolved solids (TDS) of the synthetic brine used to prepare the gels are 223.80 g/L, and that is based on the formation brine of the Tahe Oilfield. The composition of the brine is presented in Table 4.

### 4.2. Methods

#### 4.2.1. Preparation of Gel

The gelling solution is prepared from a copolymer solution, cross-linking agent, and stabilizer at room temperature. The cross-linking agent (HQ, HMTA, etc) and stabilizer (thiourea, Nano-SiO_2_) and synthetic water were weighed separately according to the predetermined gel composition, then were mixed and stirred until dissolved and mixed well. According to the predetermined gel composition, the weighed copolymer solution was added. The gelling solution was stirred at 200 rpm for 10 min. A total of 20 g of the prepared gelling solution was transferred into the ampoule bottles, and the ampoule bottles were sealed with an alcohol burner and put into a thermostatic oven to investigate the gelling properties. The thermostatic oven temperature was set at 130 °C.

#### 4.2.2. Determination of Gelation Time and Strength of Gel

The bottle test was used to qualitatively determine the gelation time and gel strength (Sydansk’s gel code method [31]). The time of the gel samples to reach the Sydansk’s code of D is called gelation time. The gel sample was sealed in an ampoule bottle to visually check its gel time and evaluate its long-term stability. In this study, the thermal stability tests were conducted at 130 °C, and the gel strength was observed at room temperature.

Since the Sydansk’s gel code method can only qualitatively evaluate the gel forming performance, it is considered to further characterize the gel strength with storage modulus. The rheological behavior of the gel sample was determined by a rheometer equipped with a temperature control unit (Anton Paar MCR92). At room temperature of 25 °C, a PP25 rotor (parallel plate geometry system with diameter of 20 mm and clearance of 1 mm) was selected, an amplitude scanning mode was adopted, and a constant frequency of 1 Hz and shear strain range of 1–500% [16,32] were set.

#### 4.2.3. Experiment Method of Plate Model

The heterogeneous plate model was placed in the oven, the temperature was kept constant at 130 °C for 12 h, and it was saturated with the simulated Tahe water at a flow rate of 0.5 mL/min. The pore volume of the plate model was calculated according to the volume of water pumped in and out as Formula (1):

*V_P_* = *Qt* − *V* (1)

where *V_P_*—pore volume of plate model, mL; *Q*—Flow of the Tahe simulation water, mL/min; *t*—Water injection time, min; *V*—Water yield, mL.

The oil was saturated at a flow rate of 0.5 mL/min until there was no water expelled. The volume of oil saturated was determined by the volume of water expelled. All the valves were closed, and the flat model was kept saturated with oil in the oven at 130 °C for 24 h. The porosity volume of the plate model was 203.1 mL, and 183 mL oil was saturated.

The valve was opened and the water was injected at 3.0 mL/min. The expelled volume of liquid and the corresponding pressure was recorded until the water content reached 94%–95%. Gelling liquid was injected and replaced with displacement liquid. All valves were closed waiting for gelation at 130 °C for 24 h, water was continually injected at 3.0 mL/min, and the expelled volume of liquid and corresponding pressure was recorded. Water injection was stopped when the water cut reached 98%. The recovery and water cut can be calculated by the produced liquids. The appearance of oil sand in the model was observed and recorded. The oil sand was divided into five parts and was taken out in blocks for dry distillation test. The remaining oil saturation of different parts was measured.

## Figures and Tables

**Table 1:** The influence of the type of phenol crosslinking agent on gel properties.

HMTA/%	HQ/%	Resorcinol/%	Phenol/%	Gelling Time/h	Storage Modulus/Pa	Time of Dehydration Rate up to 20%/d
0.1	0.1	/	/	15	3.3	70
	/	0.1	/	360	3.6	42
			0.1	38	3.5	50
	0.05	0.05	/	300	3.7	56
	0.05	/	0.05	35	3.5	46
	/	0.05	0.05	264	3.6	38
	0.08	0.02	/	96	3.7	39
	0.08	/	0.02	28	3.6	50
	/	0.08	0.02	288	3.6	33

**Table 2:** Effect of type of aldehyde crosslinking agent on gel properties.

HQ/%	P-benzaldehyde/%	M-benzaldehyde/%	HMTA/%	Gelling Time/h	Storage Modulus/Pa	Time of Dehydration Rate up to 20%/d
0.1	/	/	0.1	15	3.3	70
	0.1	/	/	No gel formed	/	/
	/	0.1	/	No gel formed	/	/

**Table 3:** Effect of deoxidizer type on gel properties.

Crosslinking Agent	Thiourea /wt%	Sodium Nitrite/wt%	Sodium Thiosulfate/wt%	Gelling Time/h	Storage Modulus/Pa	Time of Dehydration Rate up to 20%/d
0.1 wt% HQ + 0.2 wt% HMTA	0.4	/	/	13	6.2	107
	/	0.4	/	36	2.3	52
	/	/	0.4	44	4.7	58

**Table 4:** Ionic composition of synthetic water of Tahe oilfield.

Ions	Concentration, g/L
Sodium	73.30
Calcium	11.27
Magnesium	1.52
Chloride	137.53
Bicarbonate	0.18
TDS	223.80

## Data Availability

The data generated and analyzed during this study are available from the corresponding author upon reasonable request.
